# Meta-analysis of differences in Constant-Murley scores for three mid-shaft clavicular fracture treatments

**DOI:** 10.18632/oncotarget.18456

**Published:** 2017-06-12

**Authors:** Wei Jiang, Hua Wang, Yu-Sheng Li, Tian-Jian Zhou, Xin-Jia Hu

**Affiliations:** ^1^ Department of Bone and Joint, Shenzhen People’s Hospital, 2nd Clinical Medical College of Jinan University, Shenzhen 518020, China; ^2^ Department of Orthopedics, Xiangya Hospital, Central South University, Changsha 410008, China

**Keywords:** clavicular fracture, non-operative treatment, plate fixation, intramedullary pin fixation, Constant-Murley score

## Abstract

There is no consensus on the optimal treatment for mid-shaft clavicular fracture. We conducted a meta-analysis to compare the effectiveness of non-operative treatment, plate fixation, and intramedullary pin fixation in terms of the Constant-Murley Score (CMS) for treatment of mid-shaft clavicular fracture. Comprehensive search of the Embase, Cochrane Library and PubMed was conducted to retrieve relevant randomized controlled trials (RCTs). A random-effect network meta-analysis was conducted within a Bayesian framework using Markov Chain Monte Carlo (MCMC) in OpenBUGS 3.2.2. Differences in CMS among the three treatments analyzed were evaluated with weighted mean difference (WMD) and surface under the cumulative ranking curves (SUCRA). Eleven studies met our inclusion criteria and were included in our network meta-analysis. Our results revealed that in terms of CMS followed-up for six months, the efficacies of plate fixation and intramedullary pin fixation were higher than non-operative treatment (plate fixation: WMD = 4.70, 95% CI = 1.21 ∼ 7.83; intramedullary pin fixation: WMD = 6.71, 95% CI = 3.20 ∼ 10.39), and intramedullary pin fixation had better efficacy than plate fixation, had better efficacy. However, no differences were found between the efficacies of the three treatments in pairwise comparisons with respect to CMS followed-up for six weeks, three months, 12 months and 24 months. In addition, the cluster analysis showed that intramedullary pin fixation had the best efficacy for patients with mid-shaft CF, followed by plate fixation and non-operative treatment. These analyses suggest intramedullary pin fixation may be the optimal therapeutic approach for mid-shaft clavicular fracture patients.

## INTRODUCTION

The clavicle, located directly above the first rib, is one of the most commonly fractured bones, accounting for about 3%∼5% of all fractures [1]. Clavicular fracture (CF) generally occurs in the mid-shaft (81%), while lateral (17%) together with medial fractures (2%) are less frequently [2]. The incidence of mid-shaft CF is approximately 29∼64 per 100,000 persons annually; additionally, children have significantly higher rates of CF compared to adults [3, 4]. Typically, mid-shaft CF results from falls, sport injuries and motor vehicle accidents. The main reasons for mid-shaft CF are falling onto outstretched hands, a direct hit to the clavicle, and direct falls on the shoulder [5]. Non-surgical treatment options are the first line of intervention for mid-shaft CF treatment, and surgery is considered when other treatments fail, or as a corrective intervention [6]. However, serious complications can occur in mid-shaft CF patients after treatment, including nonunion or re-fracture along with malunion, which further results in chronic pain, weakness, decreased range of motion and cosmetic deformity [4]. Thus, identifying the most efficacious and safest approach for mid-shaft CF treatment would help increase the likelihood of optimal restoration of shoulder stability and function for patients [7].

Non-operative traditional treatments (mostly sling or figure-of-eight bandage) have been used to treat mid-shaft CF, even when the clavicle is substantially displaced [8]. However, studies have shown that with higher rates of nonunion, malunion, and patient dissatisfaction, the outcome of non-operative treatments is not as satisfactory as previously thought [9]. Currently, surgical options of plate fixation and intramedullary pin fixation are commonly applied in patients with mid-shaft CF [10]. Pujalte GG proposed that when compared with non-operative treatment, mid-shaft CF treated with plate fixation may lead to improved functional outcome with a lower rate of malunion and nonunion [11]. Plate fixation entails a large operative wound and stripping of soft tissue, and is associated with complications, such as nonunion, infection, wound breakdown, and local numbness with loss of reduction [12]. Recently, surgeons have attempted to use intramedullary pin fixation to treat mid-shaft CF, but this approach also yielded rates of nonunion and infection similar to those reported for plate fixation [5]. Potential limitations include hardware migration or failure, re-fracture after hardware removal, painful prominent hardware, and the development of nonunion [13]. Since no consensus has been reached on the optimal treatment for mid-shaft CF, measuring the relative efficacies of different treatments procedures could help to improve the surgical outcomes and experiences of patients [14, 15].

Traditional meta-analyses combine the results of homogeneous studies conducted on the same topic, and it is not feasible to compare more than two interventions at the same time [16]. However, a network meta-analysis can indirectly compare three or more procedures, using a common comparator, when a head-to-head trial is not available, and can also simultaneously compare several intervention methods by combining direct and indirect comparisons [17, 18]. Therefore, to help establish the optimal treatment for patients with mid-shaft CF, we performed a network meta-analysis based on previous studies to compare the efficacies of non-operative treatment, plate fixation, and intramedullary pin fixation in terms of the Constant-Murley Score (CMS).

## RESULTS

### Baseline characteristics of included studies

**Figure 1:**
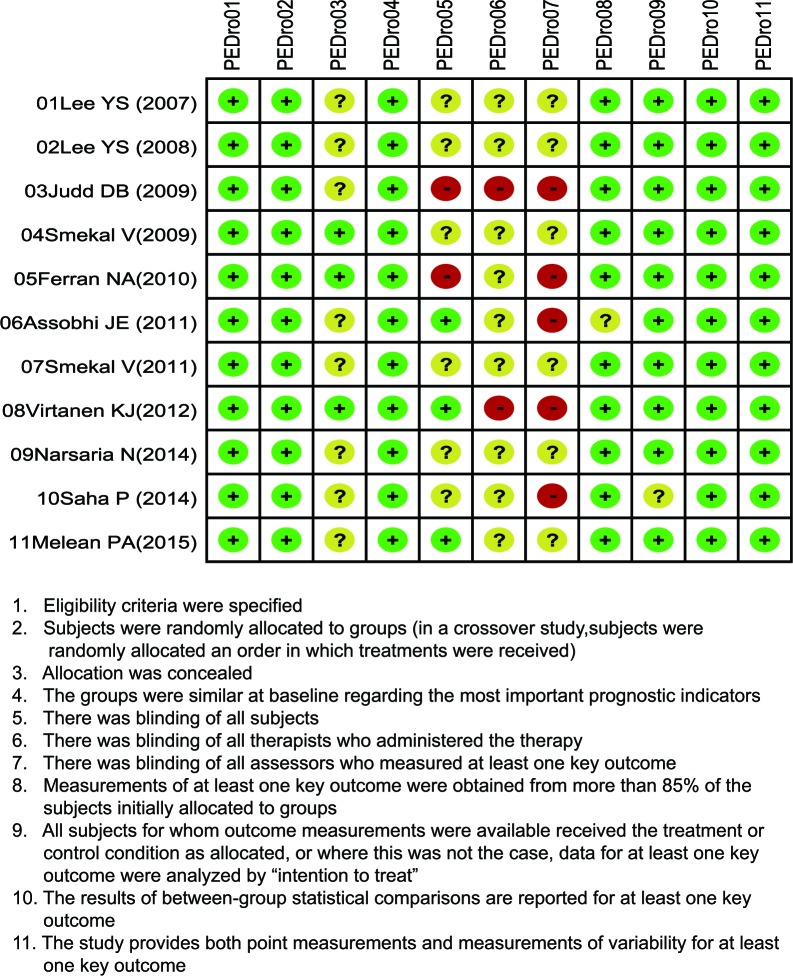
Methodological quality of assessment for included studies from the literature, based on the Physiotherapy Evidence Database (PEDro) scale

Our electronic literature search broadly identified a total of 189 potentially pertinent studies. After reading the titles and abstracts, we excluded 44 duplicates. The remaining 145 articles were further evaluated with 134 articles being removed for following reasons: 36 for monotherapy of mid-shaft CF, 53 for no relation to research topic, 21 for proceedings and abstracts, 13 for non-human studies, and one for low degree of association of outcomes investigated. In the end, a total of 11 studies, published between 2007 and 2015, met our predetermined inclusion criteria, and were thus incorporated into our network meta-analysis [1, 10, 19–27]. Taken together, these 10 studies included 721 mid-shaft CF patients (non-operative treatment: *n* = 184; plate fixation: *n* = 256; intramedullary pin fixation: *n* = 281). All of the included studies were two-arm trials, including 11 comparisons. The baseline characteristics of included studies and the PEDro scale are displayed in Table 1 and Figure 1, respectively.

**Table 1:** The baseline characteristics of ten enrolled studies

First author	Year	Country	Interventions		Total	Sample size		Gender (M/F)		Age (years)		Follow-up time(weeks/months)
			T1	T2		T1	T2	T1	T2	T1	T2	
Lee YS [19]	2007	China	B	C	62	30	32	13/17	13/19	56.7 (52–59)	60.4 (50–81)	6 m
Lee YS [20]	2008	China	B	C	88	32	56	20/12	37/19	38.2	40.1	6 m
Judd DB [10]	2009	USA	A	B	57	28	29	25/3	27/2	25 (17–41)	28 (19–40)	3 w, 6 w, 3 m, 6 m, 12 m
Smekal V [1]	2009	Austria	A	C	60	30	30	26/4	26/4	39.8 ± 14.5	35.5 ± 11.8	6 m, 24 m
Ferran NA [21]	2010	UK	B	C	32	15	17	13/2	14/3	35.4 (16–53)	23.8 (13–42)	12 m
Assobhi JE [22]	2011	Egypt	B	C	38	19	19	17/2	16/3	32.6 ± 5.9	30.3 ± 4.8	6 w, 3 m, 6 m, 12 m
Smekal V [23]	2011	Austria	A	C	112	52	60	44/8	54/6	38.0 ± 14.8	36.8 ± 12.6	24 m
Virtanen KJ [24]	2012	Finland	A	B	60	32	28	28/4	24/4	33.0 ± 12.0	41.0 ± 10.8	3 m, 12 m
Narsaria N [25]	2014	India	B	C	65	32	33	26/6	24/9	40.2 ± 11.2	38.9 ± 9.1	24 m
Saha P [26]	2014	India	B	C	71	37	34	30/7	30/4	33.32 ± 11.84	33.03 ± 12.64	6 w, 3 m, 6 m, 12 m,18 m, 24 m
Melean PA [27]	2015	Chile	A	B	76	42	34	NR	NR	37.2 ± 11.2	38.1 ± 13.0	3 m,6 m,12 m

T, treatment; M, male; F, female; NR, not report; A, non-operative treatment; B, plate fixation; C, intramedullary pin fixation; m, months; w, weeks.

### Pairwise meta-analysis

**Figure 2:**
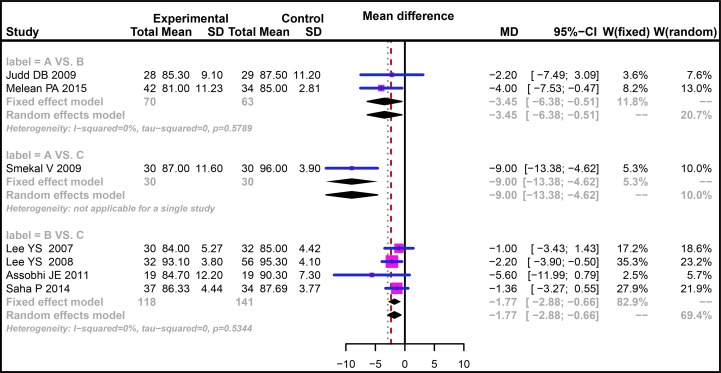
Forest plots comparing the efficacy of three treatments in patients with clavicle fracture in terms of CMS followed-up for six months (**A**) Non-operative treatment. (**B**) Plate fixation. (**C**) Intramedullary pin fixation. CMS, Constant-Murley Score.

The efficacies of three treatment methods for mid-shaft CF were evaluated with direct-paired comparisons. The results indicated that the efficacy of non-operative treatment for mid-shaft CF was relatively poor compared with plate fixation in terms of CMS followed-up for six weeks (WMD = −9.70, 95% CI = −17.65 ∼ −1.75) while no difference was found for the efficacies in comparison between plate fixation and intramedullary pin fixation (WMD = −2.00, 95% CI = −10.67 ∼ 6.68) (Supplementary Figure 1A). As for the CMS followed-up for three months, our results showed no difference in the direct paired comparisons of non-operative treatment vs. plate fixation and plate fixation vs. intramedullary pin fixation (WMD = −1.89, 95% CI = −4.37 ∼ 0.59; WMD = −1.86, 95% CI = −4.40 ∼ 0.67, respectively) (Supplementary Figure 1B). There was no obvious heterogeneity for comparisons of non-operative treatment vs. plate fixation and plate fixation vs. intramedullary pin fixation with respect to CMS followed-up for six months (*I*^2^ = 0.0%, *P* = 0.5789; *I*^2^ = 0.0%, *P* = 0.5344, respectively), thus we adopted the fixed-effect model. Additionally, the results of direct paired comparisons showed that compared with plate fixation and intramedullary pin fixation, the efficacy of non-operative treatment for mid-shaft CF was relatively poor (WMD = −3.45, 95% CI = −6.38 ∼ −0.51; WMD = −9.00, 95%CI = −13.38 ∼ −4.62, respectively), and the efficacy of plate fixation was relatively poor when compared with intramedullary pin fixation (WMD = −1.77, 95% CI = −2.88∼ −0.66) (Figure 2). For CMS followed-up for 12 months, no difference was found between plate fixation and non-operative treatment (WMD = −1.49, 95% CI = −7.95∼ 4.97), and the efficacy of plate fixation was poorer than that of intramedullary pin fixation (WMD = −2.25, 95% CI = −4.17 ∼ −0.34) (Supplementary Figure 1C). For CMS followed-up for 24 months, the efficacy of non-operative treatment was poorer than that of intramedullary pin fixation (WMD = −3.51, 95% CI = −5.05∼ −1.98), and no significant difference was found between plate fixation and intramedullary pin fixation (WMD = 0.10, 95% CI = 2.97 ∼ 3.17) (Supplementary Figure 1D).

### Pooled results of network meta-analysis

#### Evidence network

**Figure 3:**
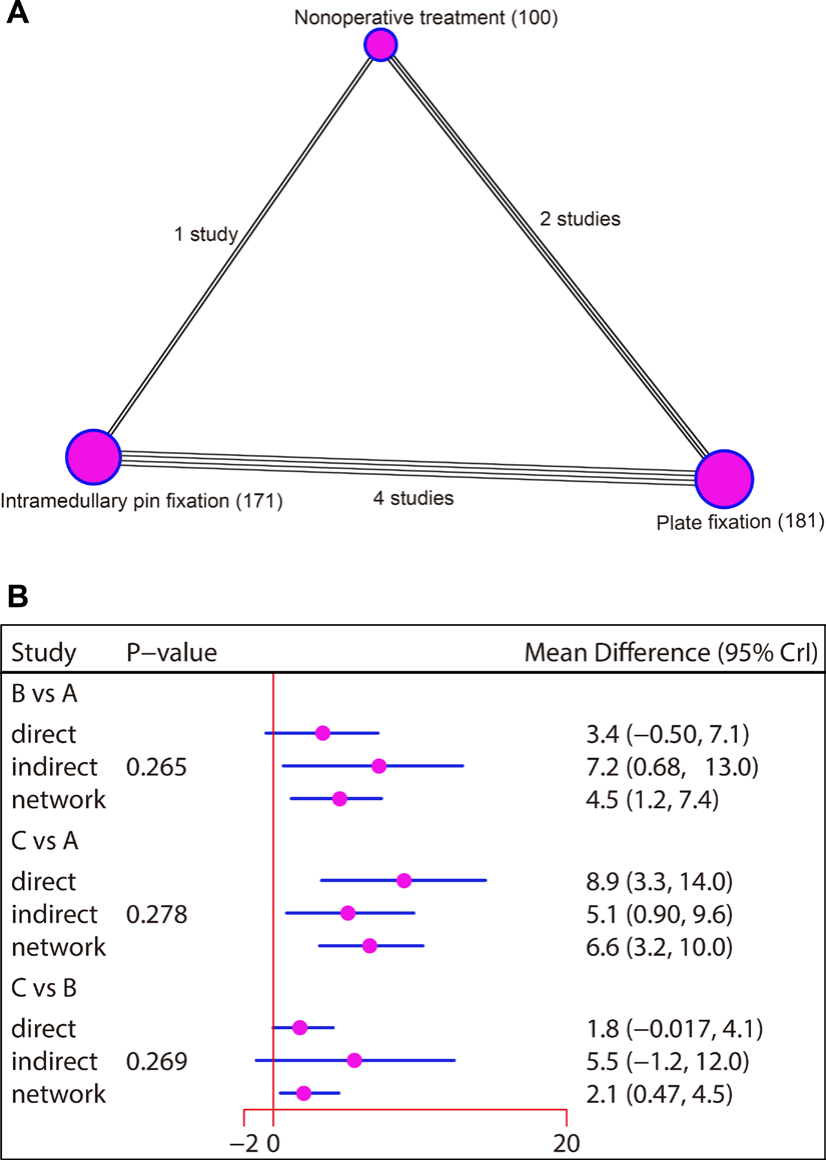
Network diagram and node-splitting method comparing the efficacy of three treatments in patients with clavicle fracture in terms of CMS followed-up for six months (**A**) Non-operative treatment. (**B**) Plate fixation. (**C**) Intramedullary pin fixation. CMS, Constant-Murley Score.

The present network meta-analysis included three kinds of treatments: non-operative treatment, plate fixation and intramedullary pin fixation. In terms of CMS followed-up for six weeks, the majority of the patients received plate fixation. In this network meta-analysis, the majority of the analyzed studies showed direct comparisons for plate fixation and intramedullary pin fixation (Supplementary Figure 2A). With respect to CMS followed-up for three months, most patients were treated with plate fixation. In this network meta-analysis, most of the analyzed studies showed direct comparisons for non-operative treatment and plate fixation (Supplementary Figure 2B). As for CMS followed-up for six months, most of the patients were treated with plate fixation. In this network meta-analysis, the majority of the studies analyzed showed direct comparisons for plate fixation and intramedullary pin fixation (Figure 3A). In terms of CMS followed-up for 12 months, the majority of patients were treated with plate fixation. In this network meta-analysis, most of the previous studies analyzed showed direct comparisons for non-operative treatment and plate fixation (Supplementary Figure 2C). With respect to CMS followed-up for 24 months, most patients were treated with intramedullary pin fixation. In this network meta-analysis, most of the studies analyzed showed direct comparisons for non-operative treatment and intramedullary pin fixation (Supplementary Figure 2D).

### Inconsistency test

The consistency model was used since the data of CMS followed-up for six weeks, three months, 12 months and 24 months were all non-network data without closed-loop. As for the network data of CMS followed-up for six months, the node partition method was used for inconsistency tests. The results showed that all direct and indirect evidence were consistent, and the consistency model was adopted (all *P* > 0.05) (Figure 3B).

### Main results of network meta-analysis

**Figure 4:**
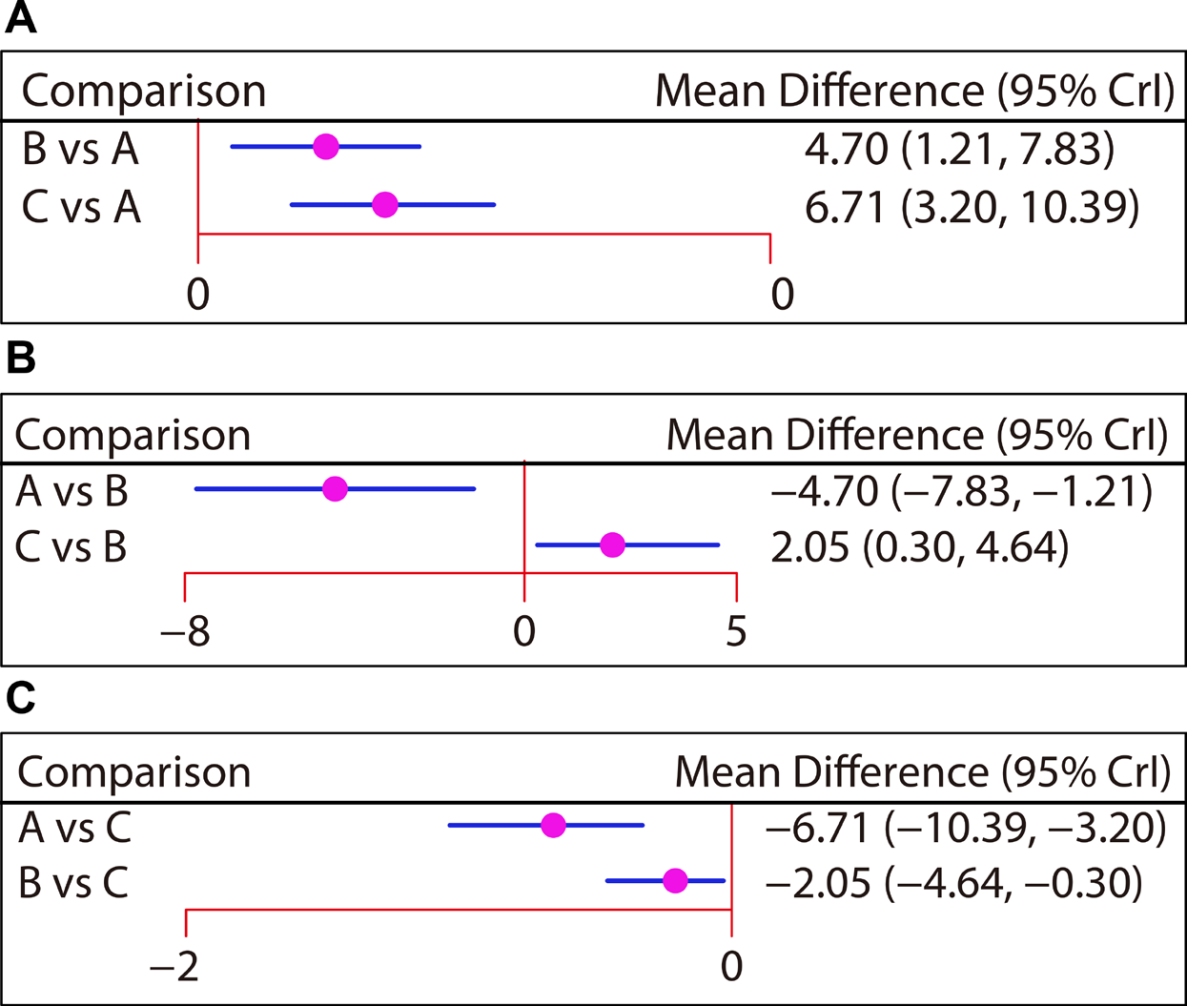
Forest plots of the relationship between efficacy comparisons for three treatments in patients with clavicle fracture in terms of CMS followed-up for six months (**A**) Non-operative treatment. (**B**) Plate fixation. (**C**) Intramedullary pin fixation. CMS, Constant-Murley Score.

Seven studies reported the efficacies of non-operative treatment, plate fixation, and intramedullary pin fixation to treat mid-shaft CF with a follow-up period of six months. Here, our network meta-analysis showed that the efficacies of plate fixation and intramedullary pin fixation were better than that of the non-operative treatment (WMD = 4.70, 95% CI = 1.21 ∼ 7.83; WMD = 6.71, 95% CI = 3.20 ∼ 10.39, respectively). Compared with plate fixation, mid-shaft CF patients treated with intramedullary pin fixation had better outcomes (WMD = 2.05, 95% CI = 0.30 ∼ 4.64) (Figure 4 and Table 2). Additionally, the efficacies of these three treatments for mid-shaft CF with follow-up periods of six weeks, three months, 12 months, and 24 months were reported in three, five, six and four studies, respectively. However, our network meta-analysis indicated that there was no difference for the efficacies of these three treatments in terms of CMS in pairwise comparisons (Supplementary Figure 3 and Table 3).

**Table 2:** Weighted mean difference and 95% confidence intervals of three treatment modalities of clavicular fractures patients in terms of the Constant-Murley Score after 6 months

WMD (95% CI)		
**Non-operative treatment**	4.70 (1.21, 7.83)	6.71 (3.20, 10.39)
−4.70 (−7.83, −1.21)	**Plate fixation**	2.05 (0.30, 4.64)
−6.71 (−10.39, −3.20)	−2.05 (−4.64, −0.30)	**Intramedullary pin fixation**

Notes: Comparison between treatments should be read from column to row. WMD, weighted mean difference; 95% CI, 95%confidence intervals.

**Table 3:** Weighted mean difference and 95% confidence intervals of three treatment modalities of clavicular fractures patients in terms of the Constant-Murley Score after 6 weeks, 3 months, 12 months and 24 months

WMD (95% CI)		
**(a) 6 weeks**		
**Non-operative treatment**	9.43 (−5.49, 24.37)	10.90 (−5.90, 29.25)
−9.43 (−24.37, 5.49)	**Plate fixation**	1.44 (−7.39, 11.90)
−10.90 (−29.25, 5.90)	−1.44 (−11.90, 7.39)	**Intramedullary pin fixation**
**(b) 3 months**		
**Non-operative treatment**	1.93 (−2.95, 7.26)	4.43 (−2.63, 13.15)
−1.93 (−7.26, 2.95)	**Plate fixation**	2.43 (−2.86, 9.15)
−4.43 (−13.15, 2.63)	−2.43 (−9.15, 2.86)	**Intramedullary pin fixation**
**(c) 12 months**		
**Non-operative treatment**	1.69 (−3.68, 6.62)	4.97 (−2.61, 12.37)
−1.69 (−6.62, 3.68)	**Plate fixation**	3.29 (−2.08, 8.62)
−4.97 (−12.37, 2.61)	−3.29 (−8.62, 2.08)	**Intramedullary pin fixation**
**(d) 24 months**		
**Non-operative treatment**	4.33 (−1.47, 9.99)	3.57 (−0.47, 7.80)
−4.33 (−9.99, 1.47)	**Plate fixation**	−0.77 (−4.75, 3.35)
−3.57 (−7.80, 0.47)	0.77 (−3.35, 4.75)	**Intramedullary pin fixation**

Notes: Comparison between treatments should be read from column to row. WMD, weighted mean difference; 95% CI, 95% confidence intervals.

### Surface under the cumulative ranking curves (SUCRA)

As shown in Table 4, the SUCRA value of intramedullary pin fixation was highest with respect to CMS followed-up for six weeks, three months, six months and 12 months (six weeks: 78.0%; three months: 88.5%; six months: 98.5%; 12 months: 91.0%). As for CMS followed-up for 24 months, plate fixation had the highest SUCRA value (86.7%), followed by pin fixation (76.7%); The SUCRA value of non-operative treatment was the lowest (six weeks: 8.5%; three months: 12.5%; six months: 0.5%; 12 months: 16.5%; 24 months: 36.7%).

**Table 4:** SUCRA values of three treatment modalities of clavicular fractures patients in terms of the Constant-Murley Score after five periods

Treatments	SUCRA values				
	6 weeks	3 months	6 months	12 months	24 months
**Non-operative treatment**	0.085	0.125	0.005	0.165	0.367
**Plate fixation**	0.635	0.490	0.500	0.430	**0.867**
**Intramedullary pin fixation**	**0.780**	**0.885**	**0.985**	**0.910**	0.767

Notes: SUCRA, surface under the cumulative ranking curves.

### Cluster analysis

**Figure 5:**
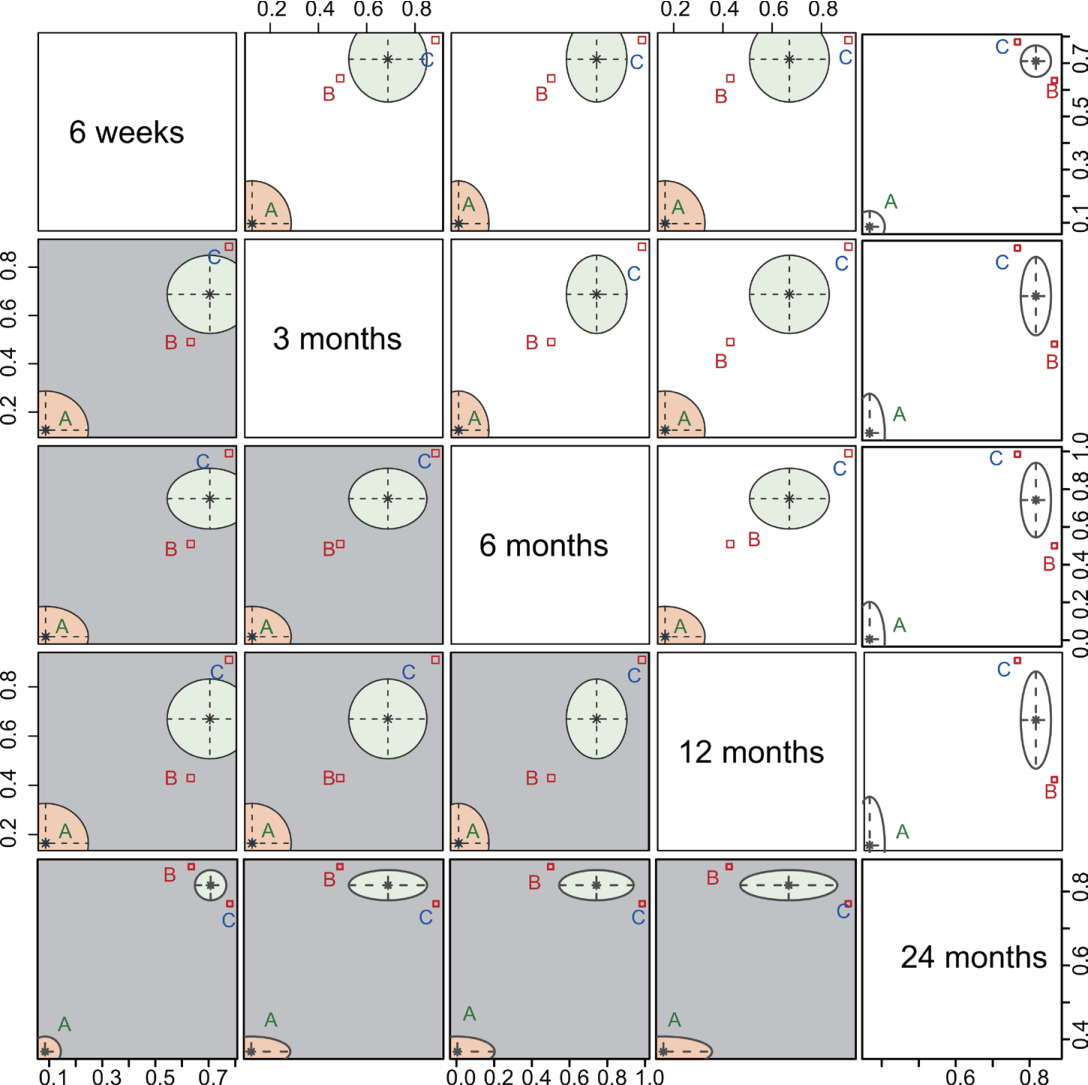
Plot of cluster analysis based on the SUCRA values with respect to CMS follow-up time The follow-up times were six weeks, three months, six months, 12 months and 24 months. (**A**) Non-operative treatment. (**B**) Plate fixation. (**C**) Intramedullary pin fixation. CMS, Constant-Murley Score; SUCRA, surface under the cumulative ranking curve.

Cluster analysis based on the SUCRA values with respect to CMS followed-up for six weeks, three months, six months, 12 months and 24 months revealed that intramedullary pin fixation had the best efficacy for patients with mid-shaft CF, followed by plate fixation and non-operative treatment (Figure 5).

## DISCUSSION

The main results of our network meta-analysis revealed that the efficacy of intramedullary pin fixation in patients with CF was better compared to that of non-operative treatment and plate fixation in terms of CMS followed-up for six months. It has been previously reported that various problems can arise when mid-shaft CF patients are treated with non-operative treatment, including 1) pain and instability in the first three weeks after injury; 2) difficulty in daily care because of multiple traumas; 3) elevated tightness of figure-of-eight bandage leading to phlebostasis of arm veins; and 4) high nonunion rate in cases of severe displacement and high-energy trauma. Thus, an alternative surgical treatment for CF was recommended [19]. Surgery is increasingly accepted as the primary treatment for CF, mainly because the outcomes of non-operative treatment are inferior [1]. As a type of operative treatment, plate fixation provides the advantage of stable bony fixation with an instrument, which is kept away from potentially risky infraclavicular structures. This poses minimal risk of implant prominence problems and results in fewer complications, early healing, and a rapid and complete return to normal function [28]. Intramedullary pin fixation, as a minimally invasive alternative for non-operative treatment, avoids many of the problems that occur during non-operative treatment [29]. Intramedullary pin fixation offers the benefits of fracture fixation with smaller incisions than traditional plate fixation, the ability to remove the implant via a similarly small incision with the patient under local anesthesia, and largely avoiding soft-tissue stripping at the site of fracture [30]. In accordance with our results, a previous study has reported better results and fewer complications with intramedullary pin fixation compared with both non-operative treatment and plate fixation [31].

Our study here also suggested that compared with plate fixation, intramedullary pin fixation had a better efficacy in treating CF patients in terms of CMS. For modern plate fixation, the application of a 3.5 mm reconstruction plating at the anteroinferior site permits adequate fixation of the lateral fragment and maximum fracture stability [32] because it’s easy to contour the plate to match the S shape of the clavicle compared to other older plates. Additionally, plate fixation has a low incidence of implant failure and nonunion, and avoids risk to the vital structures bellow the clavicle [22]. Despite these positive aspects, many complications have been reported for plate fixation in the treatment of mid-shaft CF. Usual complications include plate loosening, plate breakage, plate angulation, wound infection, nonunion and re-fracture after plate removal [33]. Even though plate fixation provides better biomechanics than intramedullary pin fixation because it is more resistant to torsional forces and bending, it involves greater exposure and extensive soft tissue stripping, which may impact fracture healing and elevate the risk of infection [34]. In contrast, a meta-analysis concluded that intramedullary pin fixation had a much lower rate of infection and nonunion than plate fixation due to lessened damages to blood supply during surgery [35, 36]. The main reason for the differences was that plate insertion causes more extensive damage to the periosteum as well as the surrounding soft tissues than intramedullary rod insertion. Furthermore, a plate might frequently require removal if inserted on the surface above the clavicle due to its gross prominence over skin [37]. All the available evidence suggests that intramedullary pin fixation is the optimal treatment for mid-shaft CF patients. Further confirming our results, the SUCRA values of most of follow-up periods showed the highest SUCRA value for intramedullary pin fixation, followed by plate fixation and non-operative treatment. We measured no differences between the efficacies of the three mid-shaft CF treatments analyzed in pairwise comparisons with respect to CMS followed-up for six weeks, three months, 12 months and 24 months. The reasons might be: 1) small sample size; 2) limited number of studies meeting our criteria; 3) six weeks and three months might be too early to compare the efficacies of three treatments analyzed; and 4), 12 months and 24 months are both long enough to allow for completely wound healing, making any difference between treatments more difficult to detect. Therefore, our conclusion needs to be further confirmed based on high-quality RCT studies with more detailed and complete information.

Indeed, our study also suffered from several limitations: (1) our network meta-analysis included only 11 studies, a limited number that might reduce the reliability of our results. However, all the studies included were consistent with the purpose of our analysis since they were RTCs with high quality. (2) Only CMS was used to measure shoulder function in CF patients; we did not consider other scoring methods due to incomplete data. (3) Complications indexes were not analyzed in our network meta-analysis. Of note, Jia Wang *et al.* conducted another network meta-analysis in terms of nonunion rate and infection rate [38]. These limitations might preclude our results from being generalized. Nonetheless, our network meta-analysis has several strong points. First, we performed a systematic and exhaustive literature search to identify all relevant trials to make sure that all available and pertinent data were included. Second, we compared interventions indirectly when no head-to-head trial existed, to more precise estimate efficacy by evaluating both direct and indirect comparisons. Third, our integration of the latest published evidence provides new insights into treatment procedures to improve shoulder function of mid-shaft CF patients.

In summary, our current network meta-analysis provides evidence that intramedullary pin fixation is the optimal treatment for CF patients among three inventions, since it improves the CMS of CF patients compared to the other two procedures analyzed.

## MATERIALS AND METHODS

### Literature search

We searched electronic literature databases using Embase, Cochrane Library and PubMed (last updated search in April 2017) to identify studies relevant to three surgical procedures in CF. A combination of keywords and free words were used to retrieve studies relevant to the topic of interest, including clavicular fracture, treatment, plate fixation and intramedullary pin fixation. We also manually searched related bibliographies to identify studies that were missed in the electronic search.

### Inclusion and exclusion criteria

The literature was systematically reviewed according to the following criteria: (1) study design: randomized controlled trail (RCT); (2) interventions: non-operative treatment, plate fixation and intramedullary pin fixation; (3) study subject: clinically and radiologically diagnosed CF patients; (4) end outcomes: the CMS. The exclusion criteria included: (1) studies with insufficient data; (2) non-RCTs; (3) duplicated publications; (4) non-human studies and (5) abstracts, systematic reviews, or meeting proceedings.

### Data extraction and quality assessment

All data from eligible trials were extracted independently by two investigators using a standard form, and any disagreements were resolved by reexamination of all items and reaching a consensus among several investigators. The methodological quality of the included RCTs was evaluated using Physiotherapy Evidence Database (PEDro) scale by two or more investigators [39]. The total scores of PEDro are 11 points, which is divided into high quality (scored ≥ four points) and low quality (scored < four points) [40].

### Statistical analysis

Firstly, we conducted traditional meta-analysis for paired comparison of direct evidence using the Meta package of R.3.2.1 software. We calculated the pooled estimates of weighted mean differences (WMDs) with corresponding 95% credible intervals (CrIs) of five periods of the CMS. We used chi-square and *I*-square tests to assess heterogeneity among the studies [41]. Afterwards, we utilized the network installation package of R.3.2.1 software to draw network diagram. In such diagrams, the nodes correspond to a variety of interventions, the node size corresponds to the sample size, and the thickness of lines corresponds to the number of enrolled studies. Additionally, gemtc installation package of R.3.2.1 software was used for network meta-analysis. In a Bayesian setting, the package gemtc offers a comprehensive set of tools to perform network meta-analysis. The four common outcome types, namely binary, continuous, count or survival, were used for the input of Arm- or contrast-level network data. As first described by Lu and Ades, gemtc modeled relative effects (*e.g*., log-odds ratio), which fitted a generalized linear model (GLM) under the Bayesian framework through connections to JAGS, OpenBUGS or WinBUGS [42]. This analysis has been further extended by others [43, 44]. In a previous study, we conducted a random-effects network meta-analysis within a Bayesian framework using Markov Chain Monte Carlo (MCMC) in OpenBUGS 3.2.2 [45]. Here, comparative WMDs are reported with their respective 95% CrIs. In our present study we utilized the node-splitting method to evaluate the consistency between direct and indirect evidence, and to select the consistency or inconsistency model based on the results [46]. To assist in the interpretation of WMDs, we calculated the probability of each treatment being the most effective or safest based on a Bayesian approach using probability values summarized as surface under the cumulative ranking curve (SUCRA). The larger the SUCRA value, the better the rank of the intervention [47, 48]. All analyses were conducted with R 3.2.1.

## SUPPLEMENTARY MATERIALS FIGURE


