# Supplementary material for: Meta-analysis of differences in Constant-Murley scores for three mid-shaft clavicular fracture treatments
Source: Oncotarget. 2017 Jun 12;8(47):83251–60. doi: 10.18632/oncotarget.18456 (PMC5669965; doi:10.18632/oncotarget.18456)
Supplement: Supplementary file 1 [file oncotarget-08-83251-s001.pdf]

Meta-analysis of differences in Constant-Murley scores for three mid-shaft clavicular fracture treatments

Supplementary Materials

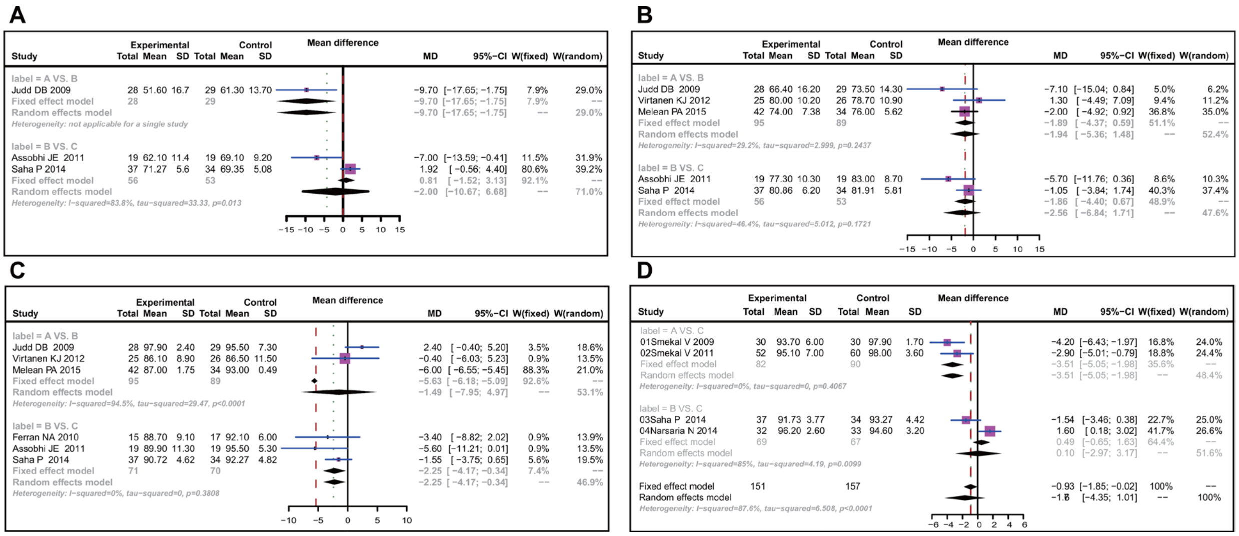

Supplementary Figure 1: Forest plots comparing the efficacy of three treatments in patients with clavicle fracture in terms of CMS follow-up time. The follow-up times were six weeks, three months, 12 months and 24 months. (**A**) Non-operative treatment. (**B**) Plate fixation. (**C**) intramedullary pin fixation. CMS, Constant-Murley Score.

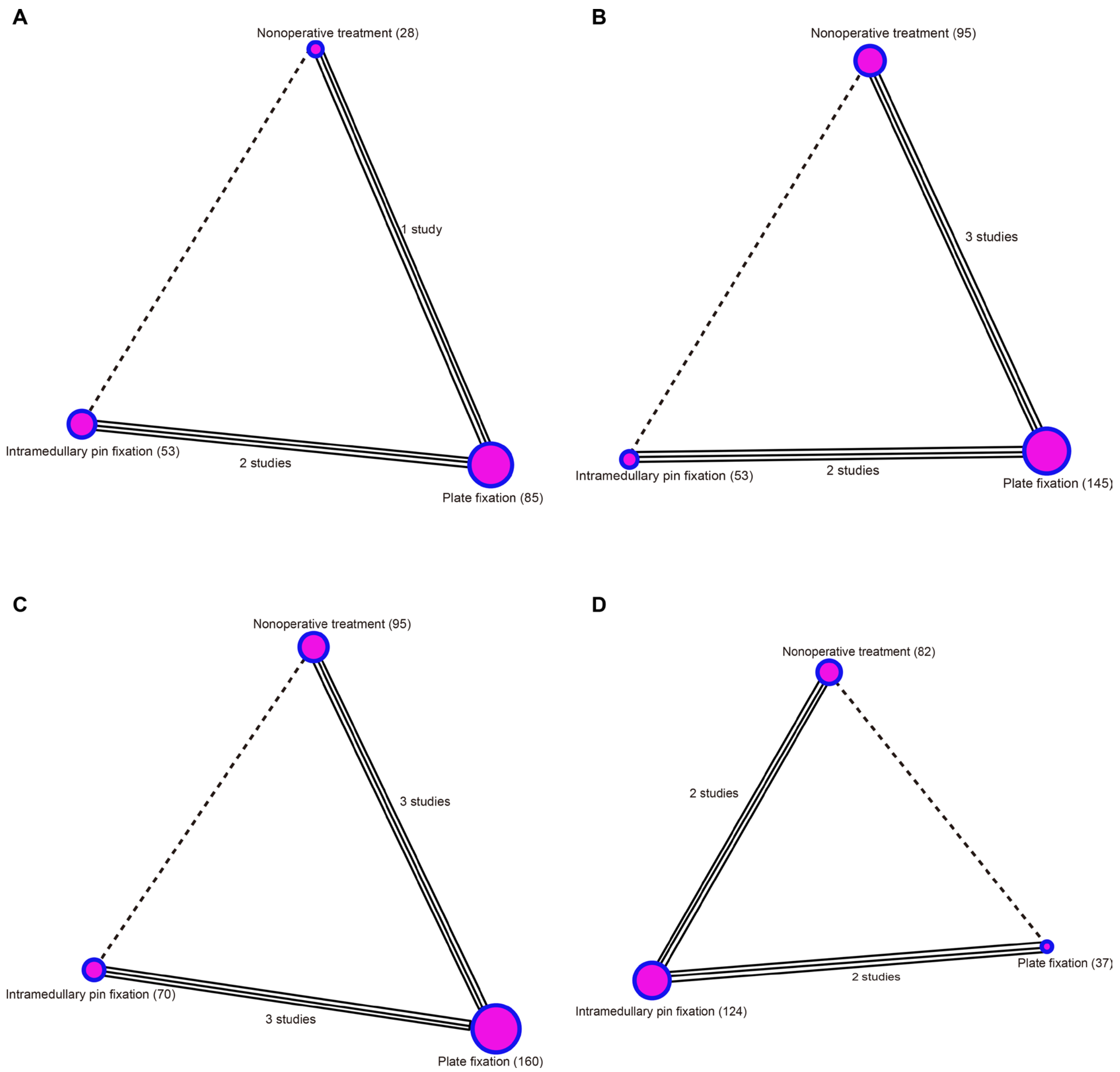

Supplementary Figure 2: Network diagram comparing the efficacy of three treatments in patients with clavicle fracture in terms of CMS follow-up time. The follow-up times were six weeks, three months, 12 months and 24 months. CMS, Constant-Murley Score.

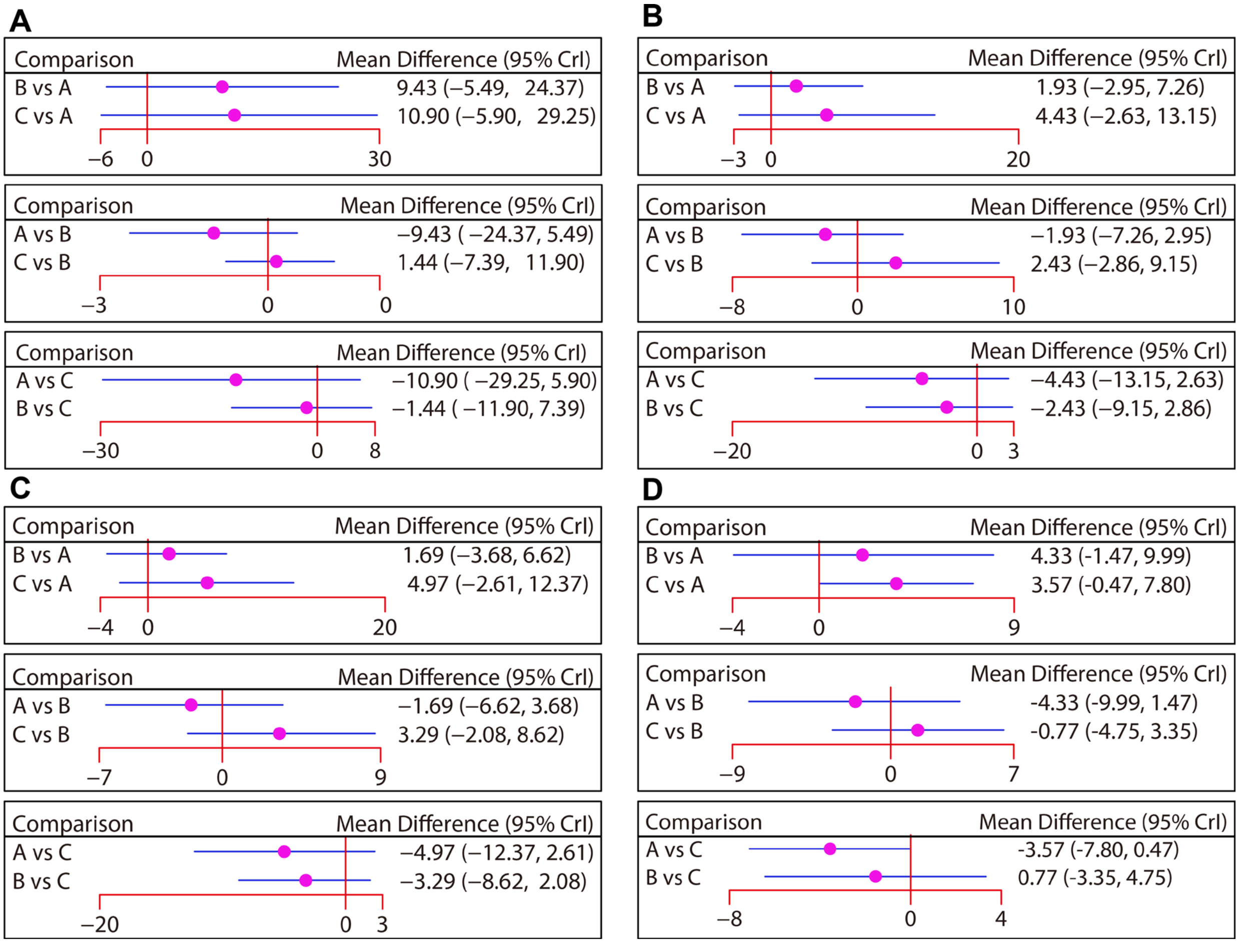

Supplementary Figure 3: Forest plots of the relationships between efficacy comparisons for three treatments in patients with clavicle fracture in terms of CMS follow-up time. The follow-up times were six weeks, three months, 12 months and 24 months. (**A**) Non-operative treatment. (**B**) Plate fixation. (**C**) intramedullary pin fixation. CMS, Constant-Murley Score.
